# RETRACTION: Sodium Hydrosulfide Relieves Neuropathic Pain in Chronic Constriction Injured Rats

**DOI:** 10.1155/ecam/9791254

**Published:** 2026-06-08

**Authors:** Evidence-Based Complementary and Alternative Medicine

RETRACTION: J. Q. Lin, H. Q. Luo, C. Z. Lin, J. Z. Chen, and X. Z. Lin, “Sodium Hydrosulfide Relieves Neuropathic Pain in Chronic Constriction Injured Rats,” *Evidence‐Based Complementary and Alternative Medicine* 2014, no. 1 (2014): 1–7, https://doi.org/10.1155/2014/514898.

The above article, published online on 25 November 2014 in Wiley Online Library (https://wileyonlinelibrary.com), has been retracted by John Wiley & Sons Ltd. The retraction has been agreed due to figure concerns initially identified on PubPeer [1]. After further investigation, further concerns were identified. Specifically:

• Figure 3 (Control) and Figure 4 (Control) appear to be the same image despite different experimental conditions
• Figure 3 (Sham) and Figure 4 (Sham) appear to be the same image despite different experimental conditions
• Figure 3 (CCI 14d) and Figure 4 (CCI) appear to be the same image despite different experimental conditions
• Figure 3 (CCI 7d) and Figure 4 (NaHSL) appear to be the same image despite different experimental conditions
• Figure 3 (CCI 21d) and Figure 4 (NaHSM) appear to be the same image despite different experimental conditions

The results and conclusions are therefore considered unreliable.

The authors did not respond when notified of the retraction.
